# Magnesium: a scoping review for Nordic Nutrition Recommendations 2023

**DOI:** 10.29219/fnr.v67.10314

**Published:** 2023-12-04

**Authors:** Christine Henriksen, Jan Olav Aaseth

**Affiliations:** 1 Department of Nutrition, Institute of Basic Medical Sciences, University of Oslo, Oslo, Norway; 2 Department of Research, Innlandet Hospital Trust, Brumunddal, Norway

**Keywords:** Magnesium, minerals, metabolism, nutrition recommendations

## Abstract

Magnesium is a divalent ion involved in a range of biochemical reactions and cellular functions. The metabolism and requirements for magnesium are still insufficiently understood. In the Nordic Nutrition Recommendations from 2012, a recommended intake was set based on balance studies. However, the average requirement (AR) was not set. Functional indicators of magnesium status have been lacking. This scoping review reveals new research activity related to the beneficial effect of magnesium intake on several health outcomes (cardiovascular disease, diabetes and some cancers). Based on meta-analyses of cohort studies and Randomized Controlled Trials (RCTs), as well as on plausible mechanisms, a causal association is suggested. However, the optimal intake cannot be set based on these study designs and no new balance studies were found.

## Popular scientific summary

- Magnesium is a mineral for the body that plays an essential role in many chemical reactions and functions inside cells.
- Whole grain cereals, milk and dairy products, vegetables, starchy roots, berries, meat, and fish are the good sources of magnesium.
- Magnesium metabolism and requirement is insufficiently understood.
- Deficiency is unusual and usually secondary to disease or use of certain medications.
- There is no qualified biomarker for magnesium status.

Magnesium is a divalent ion and is involved in a range of biochemical reactions and cellular functions. The metabolism and requirements for magnesium are still insufficiently understood. In the 2012 Nordic Nutrition Recommendations (NNR), a recommended intake was set based on balance studies (1). However, the average requirement (AR) was not set. This scoping review aims to describe the totality of evidence for the role of magnesium in health-related outcomes as a basis for setting and updating dietary reference values (DRVs) for the NNR2023 (Box 1).

Box 1 Background articles for Nordic Nutrition Recommendations 2023

- This paper is one of many scoping reviews commissioned as part of the Nordic Nutrition Recommendations 2023 (NNR2023) project (2)
- The papers are included in the extended NNR2023 report but, for transparency, these scoping reviews are also published in Food & Nutrition Research
- The scoping reviews have been peer reviewed by independent experts in the research field according to the standard procedures of the journal
- The scoping reviews have also been subjected to public consultations (see report to be published by the NNR2023 project)
- The NNR2023 committee has served as the editorial board
- While these articles are a main fundament, the NNR2023 committee has the sole responsibility for setting dietary reference values in the NNR2023 project

## Methods

This scoping review follows the protocol developed within the NNR2023 project (2), and the sources of evidence used to follow the eligibility criteria described by Christensen et al. (3). Existing systematic reviews were identified primarily by contacting major national food and health authorities and organizations, and a general web search. A literature search was conducted in MEDLINE, with the following search string inserted into PubMed, 28.2.2022: ‘magnesium’(MeSH Terms) AND (‘2011’[PDAT]: ‘3000’[PDAT]) AND review(Publication Type) AND Humans(Filter) AND (‘Diet’[All Fields] OR ‘Dietary’[All Fields] OR ‘Food’[All Fields] OR ‘Nutrition’[All Fields] OR ‘Nutritional’[All Fields])

This search string picked up 210 publications, published later than 2011. Out of these 102 articles were further scrutinized. If several reviews were (partly) based on the same original studies, only the most recent ones, with the highest quantity of studies were further evaluated. Finally, 14 publications were judged as relevant, and these are summarized in Table 1 and/or discussed in the text. Additionally, two articles (4, 5) were not picked up by the search string. References used in NNR2012 are included when relevant.

**Table 1:** Magnesium and clinical-related outcomes.

Author	Method	Population	Exposure	Outcome
**CVD**				
Zhao et al. (2019) (31)	Meta-analysis of 18 prospective studies	Healthy adults	Mg dietary intake (8 studies) Serum Mg (12 studies)	RR of CVD for the normal versus lowest serum and dietary magnesium level: 0.64 (CI 0.51.0.80)
Fang et al. (2016) (33)	Systematic review and dose-based meta-regression analysis of eight prospective studies	Healthy adults	Mg dietary intake	Dietary magnesium was inversely associated with the risk of CVD mortality (HR 0.86; 95% CI 0.81-0.92)
**Hypertension, lipids and metabolic syndrome**				
Kass et al. (2012) (34)	Systematic review and meta-analysis of 22 RCTs	Hypertensive and normotensive adults	Mg supplementation (120-970 mg/day)	Supplementation led to a decrease in SBP of 3-4 mm Hg and DBP of 2-3 mm Hg
Sarrafzadegan et al. (2016) (37)	Systematic review and meta-analysis of nine observational studies	Healthy adults and patients with MetS	Mg dietary intake (9 studies) Serum Mg (8 studies)	Dietary magnesium was inversely associated with the risk of MetS (OR 0.73; 95% CI 0.62-0.86)
**Diabetes**				
Fang et al. (2016) (38)	A Systematic Review and Meta-Regression Analysis of 25 Prospective Cohort Studies	Healthy adults and patients with type 2 diabetes	Mg dietary intake	A linear dose-response relationship was found where the risk of T2D incidence was reduced by 8%-13% for per 100 mg/day increment in dietary magnesium intake.
Simental-Mendia et al. (2016) (39)	A systematic review and meta-analysis of RCT	Healthy adults and patients with type 2 diabetes	Mg supplementation (300-1000 mg/day)	Supplementation for > 4 months improved HOMA-IR index and fasting glucose (*P* < 0.001)
**Colorectal cancer**				
Meng et al. (2019) (40)	One meta-analysis of eight cohort studies and one of three case-control studies?		Mg dietary intake	SR Ca, Mg, and K were negatively related with the occurrence of CRC.

To answer a question from one reviewer about magnesium and leg cramps, an additional literature search was conducted in MEDLINE, with the following search string inserted into PubMed, 14.12.2022: ‘magnesium’(MeSH Terms) AND (‘2011’[PDAT]: ‘3000’[PDAT]) AND (systematic review[Publication Type] OR Meta-Analysis[Publication Type]) AND Humans(Filter) AND (‘Leg cramps’[All Fields]). This search string picked up seven publications, published later than 2011. Following the same principle as described above, two publications (6, 7) were finally judged as relevant and included in the text.

## Physiology

Magnesium is a metal in group 2 in the periodic table of elements. It occurs as free Mg^2+^ cation in solutions or as the mineral part in a variety of compounds. The content of magnesium in the body is regulated by absorption and excretion. Magnesium is absorbed in ionized form, preferably in the ileum, through a paracellular process. The percentage absorbed is inversely proportional to the amount of magnesium ingested. At normal dietary intakes 40–50 % is absorbed. Inorganic forms appear to be less bioavailable than organic ones (8). A diet high in phytic acid and phosphate will reduce the absorption, but the clinical relevance of this is uncertain (5).

Plasma magnesium concentrations are kept within a narrow range of 0.75–0.95 mmol/L. When plasma concentration is low, kidney excretion is reduced. At higher concentrations, the excretion is increased. Excretion is also increased by hypernatraemia and metabolic acidosis (9), unregulated diabetes, and alcohol consumption (10).

The total body content of magnesium in an adult is estimated to be 20–28 g with 40–45% being intracellular in muscles and soft tissue, 1% being extracellular, and the remainder being found in the skeleton. Although humans do not have a true storage organ for magnesium, approximately one-third of skeletal magnesium is in equilibrium with plasma magnesium levels and functions as a buffer to maintain the physiological extracellular magnesium concentrations.

Magnesium is a cofactor in more than 500 enzymes; thus, a large number of biochemical and physiological processes are regulated by magnesium. Magnesium is necessary for energy metabolism and glucose transport across cell membranes, sustained electrical potential in nerves and cell membranes, and for transmission of neuromuscular impulses (4).

Magnesium depletion is unusual in the absence of dietary restriction and some disorders causing magnesium loss from the body. Magnesium depletion is usually secondary to another disease process or to the use of certain therapeutic agents, for example some diuretics. The physiological manifestations of severe magnesium depletion include hypokalaemia and hypocalcaemia, neuromuscular hyperexcitability, electrocardiographic abnormalities, cardiac arrhythmias and eventually cardiac arrest. Adverse heart rhythm changes have been observed in healthy women after magnesium depletion due to suboptimal daily magnesium intake in the range 101–130 mg (11).

Therapeutic use of magnesium in heart arrhythmia conditions (12) and to reduce the risk of eclampsia in women with pre-ecampsia (13) has received wide scientific attention in recent years. The neuroprotective role of antenatal magnesium sulphate therapy given to women at risk of preterm birth has also been established (14). Magnesium supplements are also given in the management of refeeding syndrome (15) and acute alcohol abstinence (16).

## Assessment of nutrient status

Assessment of magnesium status is difficult, because of the distribution in the body, where most is found in the skeleton, and less than 1% in the extracellular compartment. Measurement of magnesium in plasma/serum will thus not always reflect the intracellular level. Magnesium research has for a long time been hampered by the lack of good biomarkers of magnesium status in the body. Although magnesium is the cofactor of many enzymes, no adequate functional biomarker has been identified (17).

Concentrations of magnesium in urine, faeces, saliva, plasma/serum, and erythrocytes have been explored for assessment of magnesium status. In a systematic review, a total of 20 potential biomarkers of magnesium status were assessed from 21 included publications. Plasma/serum, red blood cells and urine concentrations responded to dietary changes and appeared to be the most useful biomarker candidates. For the other parameters, it was not possible to draw any conclusions about their usefulness as magnesium status biomarkers. There are however limited data available and subgroup analyses for sex and age were not possible to perform (17).

The European Food Safety Authority (EFSA) states that all proposed biomarkers have limitations, and considers that there are no appropriate biomarkers for magnesium status (5). Low concentrations of magnesium in plasma/serum (<0.75 mmol/l) are however useful to identify severe deficiency. Individual levels between 0.75 and 0.85 mmol/l may be suboptimal and need further evaluation (18). In clinical practice, plasma magnesium is frequently monitored in patients with malabsorption and during refeeding after malnutrition. Plasma levels are also monitored in patients with renal insufficiency due to the risk of hypermagnesemia.

## Dietary intake in Nordic and Baltic countries

Whole grain cereals, milk and dairy products, vegetables, starchy roots, berries, meat and fish are the best sources of magnesium in a mixed diet (19). Magnesium concentrations are especially high in dark chocolate, nuts, and coffee. The average dietary intake of adults in Europe ranges from 250 to 300 mg/d in females and 320–440 in men (5). ‘Hard’ water contains more magnesium than ‘soft’ water, and drinking water can contribute to 10–20 mg/day of the total magnesium intake.

The intake in the Nordic and Baltic countries are similar to the European data, the average dietary intake ranges from 260 to 350 mg/day in females and in 330–440 in males (20).

## Health outcomes relevant for Nordic and Baltic countries

Epidemiological studies have previously reported a relationship between low magnesium intake and increased risk of cardiovascular disease, hypertension, stroke, type 2 diabetes and colorectal tumour risk (21–30). However, in the 2012 revision of NNR, the total evidence was considered as weak, and high quality randomized controlled trials were requested.

### Cardiovascular disease

From the present literature search, the two most recent systematic reviews and meta-analyses regarding cardiovascular-related outcomes in healthy adults (31, 32) were included. Both were based on prospective cohorts, one with CVD and the other with CVD-mortality as the outcome.

The meta-analysis of Zhao et al. (31) was based on 18 prospective studies in healthy adults. Zhao et al. concluded that higher dietary magnesium and serum magnesium was both inversely related to total CVD risk including coronary heart disease events. The evidence supported a linear model in the range of magnesium intake 173–457 mg/day, RR 0.64 (CI 0.51.0.80) of CVD for the normal versus lowest serum and dietary magnesium level. In the analyses, most data sets were controlled for age and BMI, but the authors noticed that a lack of control for all potential confounding factors represented a limitation.

Fang et al. (33) have explored the association with cardiovascular mortality, based of eight prospective studies. Overall, an inverse association between dietary magnesium and the risk of CVD mortality (HR 0.86; 95% CI 0.81–0.92) was found. The results were adjusted for age and BMI. Notably, a dose-response effect was only present in women.

### Hypertension, lipids and metabolic syndrome

Several systematic reviews have addressed the effect of magnesium supplements on blood pressure lipids and metabolic syndrome. The four most recent, conducted in different populations, are included in this review (34–37).

Kaas et al. (34) conducted a meta-analysis of 22 RCTs in hypertensive and normotensive adults. In these studies, the intervention lasted from 3 to 24 weeks, and the magnesium supplementation was in the range of 120–970 mg/day. The supplementation led to a significant decrease in diastolic blood pressure (DBP) (effect size 0.36; 95 % CI 0.27–0.44) and systolic blood pressure (SBP) (effect size 0.32; 0.23–0.41). The effect size represented a decrease in DBP of 2–3 mm Hg and SBP of 3–4 mm Hg, which increased further with increasing supplementation dosage >370 mg. Notably, the highest dosages (970 mg magnesium) represent pharmacologic intake that cannot be achieved from diet alone.

Evidence from interventions in type 2 diabetic patients may not be applicable to the general population, butsupports tothe total evidence for an effect of magnesium supplementation on blood pressure (35). A small beneficial effect on LDL-cholesterol but not on HDL-cholesterol or triglycerides was also seen in a meta-analysis of studies on magnesium supplementation in diabetic patients (35).

Elevated blood pressure is a characteristic sign of metabolic syndrome (MetS). Sarrafzadegan et al. (37) did a meta-analysis of nine studies regarding the association between magnesium dietary intake, as well as magnesium serum levels and risk of MetS. The analysis showed that a higher intake of magnesium was associated with lower risk of MetS (OR 0.73; 95 % CI 0.62–0.86), but the association between serum magnesium and MetS was heterogeneous. The evidence from this SR is limited due to most studies being cross-sectional.

### Diabetes

Two meta-analyses have addressed the association between magnesium intake and risk of diabetes. Fang et al. (38) did a meta-regression analysis of 25 prospective cohort studies, with a median magnesium intake ranging from 115 to 478 mg. The analysis showed that the risk of type 2 diabetes (T2D) incidence was reduced by 8–13% for per 100 mg/day increment in dietary magnesium intake, after adjustment for age and BMI. Notably, only two of the studies were conducted among Europeans (Italy and Germany). Simental-Mendia et al. (39) conducted a systematic review and meta-analysis of RCT of the effect of magnesium on several markers of glucose tolerance. Studies on healthy and diabetic subjects were included. Magnesium supplements were given in dosages from 300 to 1000 mg/day. The results showed that magnesium supplementation significantly improved HOMA-IR index (10 studies) and fasting glucose in studies with duration >4 months (subgroup analysis of 22 studies). The mechanism of action is assumed to be by magnesium stimulating the tyrosine-kinase activity of the insulin receptor, but no significant effect was seen on insulin concentration or HbA1C.

Despite of this new knowledge, recommendation of magnesium supplementation to prevent T2D seems premature. Question that remains to be answered also relates to the optimal type, dose and duration of magnesium-supplementation. One concern is the increased frequency of gastrointestinal pain, including diarrhoea, frequently associated with of supplements >500 mg magnesium. Other possible risks include impaired renal clearance in patients with chronic kidney disease. An analysis of the costs (including adverse events) versus beneficial effect compared with established treatment options remains to be done.

### Colorectal cancer

Meng et al. (40) has explored the association between intake of several minerals and the risk of colorectal cancer (CRC). The authors did a meta-analysis of eight cohort studies and three case-control studies. Intake of magnesium was negatively associated with risk of CRC in the cohort studies (HR 0.80; 95% CI 0.73–0.87) and the case-control-studies (HR 0.80; 0.63–0.98). The proposed mechanism is related to the role of magnesium in maintenance of gastrointestinal and hormonal function.

### Other outcomes

Muscle cramps are common and often associated with exercise, pregnancy or ageing. This type of leg cramps is regarded as idiopathic, but magnesium supplements are marked for prophylactic use. A Cochrane systematic review of 11 studies (6) found no significant beneficial effect of magnesium supplements regarding number of cramps, intensity or duration in older adults. Oral magnesium supplementation was associated with minor adverse events, mostly of gastrointestinal nature (e.g. diarrhoea) in 11 to 37% of participants in the intervention groups. No RCT regarding exercise-associated muscle cramps were identified. The literature regarding pregnancy associated leg cramps, were conflicting (6).

Another meta-analysis of four articles examined pregnancy-associated leg cramps only (7) and included three of the same studies as the Cochrane-review. Many limitations, related to small sample sizes and heterogeneity in measurement of the endpoints were identified, and further research is needed. However, so far, there is no evidence that magnesium supplementation decreases the frequency of leg cramps in pregnancy.

A high magnesium intake has also been linked with decreased risk of mental disorders and polycystic ovary syndrome, increased muscle fitness in the elderly and bone mineral density (41–44). Because of limited number of studies and conflicting results, further research is needed to establish evidence.

### Toxicity

No upper level was stated in NNR2012. It is well known that excessive magnesium intake (0.5–5 g/day) gives diarrhoea, but otherwise no negative symptoms are observed when kidney function is normal. However, for people with renal insufficiency, magnesium supplements should be avoided. The U.S. Food and Nutrition Board (45) has set a Tolerable Upper Intake level (UL) of 350 mg magnesium/day from supplements. This level is based on the lowest observed adverse effect levels. The EU Scientific Committee for Food (46) has derived a maximum daily intake of 250 mg based on similar data. The UL does not include magnesium normally present in foods and beverages.

When taken together, the evidence suggests a causal relationship between magnesium intake and reduced risk of CVD, hypertension, MetS and improvement of glucose tolerance. There are however some limitations. In the observational studies, data on magnesium intake are taken from food frequency questionnaires. These questionnaires collect data on the usual food intake, and do not include magnesium from drinking water or food supplements. Furthermore, it is difficult to separate the effect of various components, for example fibre and magnesium because both are found in whole grain legumes and vegetables. The optimal intake of magnesium cannot be set solely based on these studies.

## Requirement and recommended intake

*Adults*. In the absence of functional indicators of magnesium status, the only basis available for evaluating an AR is balance studies. Data from 27 balance studies were pooled by Hunt and Johnson in 2006 (47) at the U.S. Department of Agriculture, and they suggested that the previously estimated AR (EAR) by the U.S. Food and Nutrition Board might have been too high. Neutral magnesium balance was predicted at a magnesium intake of 165 mg/day, and neither age nor sex affected the relation between magnesium intake and output (47). Data were reported for adults only. No new balance studies were identified since 2012.

Because absorption of magnesium varies with the dietary intake, it seems possible to adapt to a low intake through more effective absorption. The U.S. Food and Nutrition Board set an EAR for magnesium of 255 mg/day for women and 330 mg/day for men aged 19–30 years. The Recommended Dietary Allowance (RDA) is 310 and 400 mg/day for women and men, respectively. For adults aged 31–70 years the RDA for women is set at 320 mg/day and for men at 420 mg/day in this age group.

The EFSA panel concludes that AR and recommended intakes (RI) cannot be set for European population, and defines adequate intakes (AI) of 350 mg for men and 300 mg for women based on average intake in healthy populations in the European Union (5). These values are however very similar to the ones published in the NNR2012 based on balance studies.

### Infants, children and pregnancy

Data that could contribute to the development of evidence-based dietary recommendations for subgroups of the population groups such as infants, children and adolescents, and pregnant women are limited (17). Still, based on the available studies, consumption of magnesium-rich foods in line with the current food-based dietary guidelines seems advisable. Such foods are milk, whole grain cereals, starchy roots and vegetables, legumes and nuts. The magnesium content in human breast milk is 23–47 mg/L (30), and this concentration is relatively constant during the first 12 months of lactation (31).

## Conflict of interest and funding

The authors have not received any funding or benefits from industry or elsewhere to conduct this study.
